# Gangliocytic Paraganglioma of the Duodenum: A Masquerader

**DOI:** 10.31486/toj.23.0010

**Published:** 2023

**Authors:** Vishu Jain, B Selvakumar, Vaibhav Kumar Varshney, Vikarn Vishwajeet, Sameer Taywade, Lokesh Agarwal, Taruna Yadav, Rakesh Pandey

**Affiliations:** ^1^ Department of Surgical Gastroenterology, All India Institute of Medical Sciences, Jodhpur, Rajasthan, India; ^2^ Department of Pathology and Laboratory Medicine, All India Institute of Medical Sciences, Jodhpur, Rajasthan, India; ^3^ Department of Nuclear Medicine, All India Institute of Medical Sciences, Jodhpur, Rajasthan, India; ^4^ Department of Radiodiagnosis, All India Institute of Medical Sciences, Jodhpur, Rajasthan, India

**Keywords:** *Duodenal neoplasms*, *paraganglioma*, *robotic surgical procedures*

## Abstract

**Background:** Gangliocytic paraganglioma (GP) is a rare tumor that most commonly arises from the duodenum and is characterized pathologically by 3 cell types: epithelioid, spindle, and ganglion cells. GP is often difficult to differentiate from a neuroendocrine tumor on the basis of preoperative imaging, and the diagnosis is based on final histopathologic and immunohistochemical analysis.

**Case Report:** We report the case of a 28-year-old male who presented with pain in the abdomen, bilious vomiting, and weight loss. Imaging showed a mass involving the first and second part of the duodenum that was likely a neuroendocrine or gastrointestinal stromal tumor. He underwent robotic-assisted pancreatoduodenectomy, and the final pathology report identified GP with lymph node metastasis. The patient was doing well at 1-year follow-up.

**Conclusion:** GP is often a histologic surprise as most cases are diagnosed in postoperative histopathology. While GP has a more benign course than a neuroendocrine tumor, radical surgical resection is warranted in cases of diagnostic dilemma, suspicion of malignancy, or lymph node metastasis. Robotic-assisted pancreatoduodenectomy is a feasible option.

## INTRODUCTION

Gangliocytic paraganglioma (GP) is a rare tumor that arises from the duodenum in approximately 90% of cases.^1,2^ Dahl et al first described GP in 1957 as duodenal ganglioneuroma.^3^ In 1962, Taylor and Helwig presented a case series of 9 patients with tumors of similar morphology arising from the second portion of the duodenum and named them benign nonchromaffin paragangliomas because of the presence of nests of epithelioid cells.^4^ In 1971, Kepes and Zacharias first described these tumors as GP because of the presence of both ganglion cells and epithelioid cells.^5^ GPs are rare tumors, with approximately 280 cases identified in a MEDLINE search through November 2022.^2,6,7^ GP occurs most commonly in the duodenum and is indistinguishable from a neuroendocrine tumor on imaging.^8^ However, the natural history of the tumors is different, with GP having a more benign course and a better prognosis than neuroendocrine tumor.

We present the case of a 28-year-old male who presented with pain in the abdomen. A mass in the first and second part of the duodenum had differentials of neuroendocrine tumor and gastrointestinal stromal tumor, but postoperative histopathology and immunohistochemistry were suggestive of GP. To our knowledge, this case is the first report of GP managed with robotic-assisted pancreatoduodenectomy.

## CASE REPORT

A 28-year-old male presented with vague upper abdominal pain for the prior 4 years and large-volume bilious vomiting after meals for the prior month. He reported weight loss of 10 kg during the prior month. His physical examination was unremarkable. Esophagogastroduodenoscopy showed an infiltrative mucosal lesion at the junction of the first and second sections of the duodenum (D1-D2), and biopsy showed a few benign spindle cells without evidence of malignancy.

**Figure 1.**
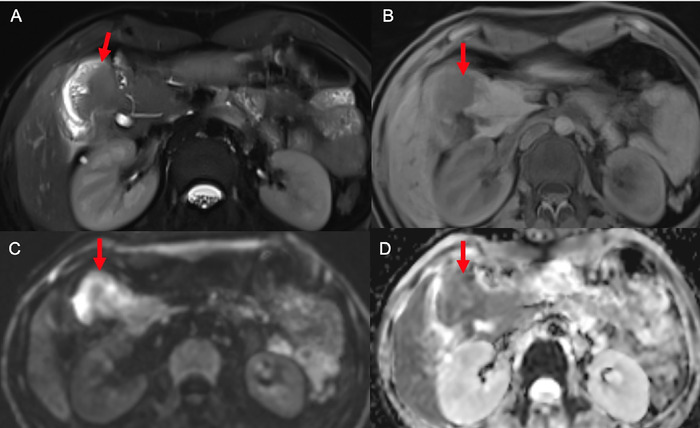
Magnetic resonance imaging of the upper abdomen. Duodenal mass (red arrows) appears isointense on (A) axial T2-weighted image and hypointense on (B) T1-weighted image. Diffusion restriction is seen in (C) diffusion-weighted image and corresponding (D) apparent diffusion coefficient map.

**Figure 2.**
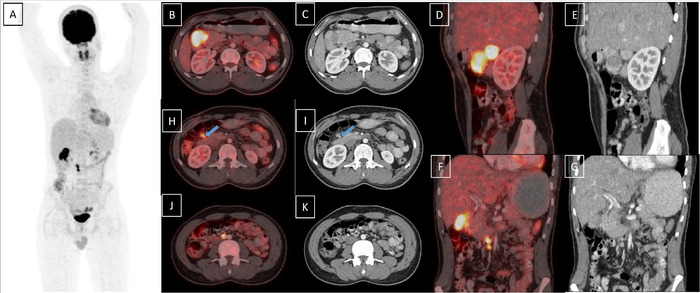
Positron emission tomography with 2-deoxy-2-(fluorine-18)fluoro-D-glucose integrated with computed tomography (18F-FDG PET-CT). (A) Maximum intensity projection image shows focal FDG uptake in the subhepatic region and a few small foci of increased uptake in the abdomen in the midline. (B, D, and F) Fused PET-CT and (C, E, and G) CT images reveal intensely FDG-avid heterogeneously enhancing lesion along the medial wall of the first and second part of the duodenum. A few FDG-avid discrete metastatic lymph nodes are also seen in axial fused and CT images involving (H and I) the hepatoduodenal region (blue arrows) and (F, G, J, and K) the aortocaval region.

Contrast-enhanced computed tomography (CT) of the abdomen suggested a 4 × 2 × 3-cm well-defined, heterogeneously enhancing mass arising from the medial wall of the D1-D2 junction with a ∼2-cm lymph node in the pancreaticoduodenal groove. The imaging differentials were duodenal neuroendocrine tumor and gastrointestinal stromal tumor. Magnetic resonance imaging (MRI) of the upper abdomen showed similar findings, with diffusion restriction in both the mass and the lymph node, suggesting a neuroendocrine tumor with lymph node metastasis (Figure 1). Positron emission tomography with 2-deoxy-2-(fluorine-18)fluoro-D-glucose integrated with CT (18F-FDG PET-CT) showed an intensely FDG-avid mass lesion along D1-D2, with maximum standardized uptake value (SUVmax) of 22.9. In addition, a few FDG-avid discrete hepatoduodenal and aortocaval lymph nodes were noted. The overall features suggested the possibility of gastrointestinal stromal tumor (Figure 2). The patient's carcinoembryonic antigen level was 1.429 ng/mL (reference range, <4 ng/mL), CA 19.9 was 4.922 U/mL (reference range, <19 U/mL), and serum chromogranin was 37.55 ng/mL (reference range, 25-140 ng/mL).

**Figure 3.**
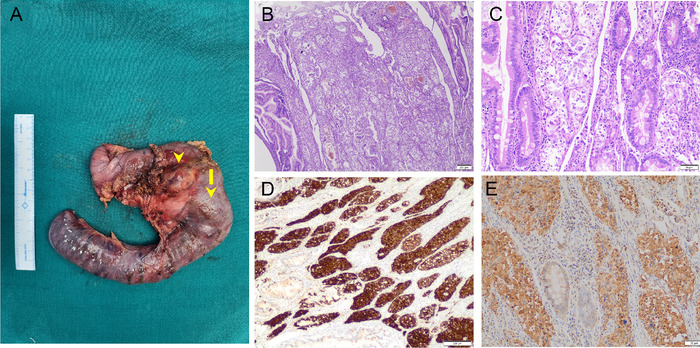
(A) Resected pancreaticoduodenectomy specimen showing the bulky duodenal tumor (arrow) and the enlarged pancreaticoduodenal lymph node (arrowhead). (B) Low-power magnification shows tumor cells arranged in clusters and infiltrating the lamina propria and submucosa (hematoxylin and eosin stain [H&E], ×20 magnification). (C) Tumor clusters include epithelioid cells, spindle cells, and occasional ganglion cells (H&E, ×100 magnification). (D) Tumor cells are positive for synaptophysin (×40 magnification) and (E) neuron-specific enolase (×10 magnification).

The patient underwent robotic-assisted pancreatoduodenectomy with placement of a feeding jejunostomy. Intraoperatively, a tumor was located in the D1-D2 region with an enlarged, firm lymph node of ∼2 cm in the pancreaticoduodenal groove (Figure 3).

Postoperatively, the patient developed a biochemical leak and superficial surgical site infection (SSI). On postoperative day (POD) 3, drain fluid amylase (DFA) of the left and right abdominal drain was 36,590 IU/L and 2,246 IU/L, respectively. Right drain output decreased gradually; on POD 6, DFA was 54 IU/L, so the right drain was removed on the same day. On POD 6, left drain output was serous in character and <50 mL, but the DFA was still high (504 IU/L). The SSI occurred in the incision from which the specimen was retrieved. The clips from the wound were partially removed, and pus was evacuated and sent for culture and sensitivity. The culture grew *Escherichia coli*, so a sensitivity-based injection of piperacillin-tazobactam 4.5 g every 8 hours for 7 days was prescribed, and daily dressing was performed. Contrast-enhanced CT of the abdomen on POD 8 revealed no significant intra-abdominal collection.

The patient was managed conservatively with nutritional support (high-protein diet) orally and via feeding jejunostomy. With continued supportive management, he improved and was discharged on POD 9 in stable condition with the left drain in situ.

At his follow-up visit to the outpatient clinic 7 days after discharge, drain output was minimal, and SSI was improved. The drain was removed, and primary closure of the surgical site was done.

Final histopathology showed a well-circumscribed tumor arising from the submucosal region of D2, reaching the muscularis propria and infiltrating the pancreas. The tumor was predominantly composed of spindle to epithelioid cells arranged in a nested and trabecular pattern with scattered ganglion cells. Microcalcification was noted. No areas of necrosis or lymphovascular or perineural invasion were seen. Immunohistochemistry showed tumor cells diffusely positive for neuron-specific enolase and synaptophysin (Figure 3). Of the 26 lymph nodes sampled, 5 were positive. The 2 lymph nodes harvested from the aortocaval and hepatoduodenal regions were free of tumor.

The patient was followed every 3 months with routine clinical examination and hematologic and biochemical investigations; findings were essentially normal. Surveillance contrast-enhanced CT of the abdomen 6 months after surgery showed no recurrent or residual disease. The patient was doing well and had no recurrence at 1-year follow-up.

## DISCUSSION

GP, an uncommon entity commonly misdiagnosed as a neuroendocrine tumor,^8^ occurs in both males and females, with slight male predominance (1.51:1) and a mean tumor size of approximately 2.57 cm^2^. The most common clinical presentation is gastrointestinal bleeding; other symptoms include abdominal pain, anemia, nausea, weight loss, fatigue, and jaundice.^2^ Our patient presented with partial gastric outlet obstruction features on the background of long-standing abdominal pain. GP can occasionally have lymph node (11.4%) or liver (1.1%) metastasis at the time of presentation.^2^

Radiologically, GP usually presents a diagnostic dilemma, similar to our case, as the contrast-enhanced CT and MRI of the abdomen showed an enhancing duodenal mass with differentials of neuroendocrine tumor and gastrointestinal stromal tumor. FDG PET-CT showed an FDG-avid lesion with high SUV uptake and lymphadenopathy, favoring gastrointestinal stromal tumor or poorly differentiated neuroendocrine tumor. On contrast-enhanced CT, GP can present as a polypoid smooth-surfaced intraluminal mass, as an area of soft tissue attenuation with a homogenously enhancing mural or extrinsic tumor present on the medial or lateral wall of the duodenum, or as a pedunculated intraluminal mass.^9^ Further, GPs are hyperintense on T2-weighted sequence and hypointense on T1-weighted sequence, similar to neuroendocrine tumors.^9^ The FDG PET-CT shows a solid lesion with FDG uptake.^10^ Thus, the radiologic findings are not specific but should raise suspicion for GP.

On pathologic analysis, GP shows 3 distinct cell types—epithelioid, spindle, and ganglion—with varying proportions. The epithelioid cells are endodermally derived and originate from the ventral primordium of the pancreas, while the ganglion cells and spindle cells are neuroectodermal in origin.^11^ On immunohistochemistry analysis, all 3 subtypes are positive for neuron-specific enolase, synaptophysin, CD56, and chromogranin A (occasional in spindle cells). The epithelioid component is also positively immunoreactive for the progesterone receptor, pancreatic polypeptide, somatostatin, and cytokeratins. The spindle cells are also positively immunoreactive for S100, neurofilament, and vimentin. The ganglion-like cells also have positivity for somatostatin, neurofilament, pancreatic polypeptide, and S100.^2^ Most GPs can be diagnosed with neuron-specific enolase immunopositivity as it is found in all 3 cell types. Other commonly used immunomarkers are synaptophysin and S100.^7,12^

Preoperative diagnosis of GP of the duodenum is often difficult. In a systematic review by Okubo et al, preoperative or endoscopic treatment biopsy was available in 63 cases, but only 12 (19%) were correctly diagnosed as GP.^2^ Six of the 63 patients were diagnosed with neuroendocrine tumor grade 1, 2 with paraganglioma, 1 with ganglioneuroma, and 1 with atypical cells. For 41 patients, the biopsy showed no tumor cells. The positive reactivity of the GP epithelioid cells for progesterone receptor and pancreatic polypeptide can differentiate GP and neuroendocrine tumor grade 1.^13^ Gastrointestinal stromal tumor, another differential of GP, can be easily differentiated in immunohistochemistry analysis because it is immunopositive for DOG-1, CD117, and CD34.^12^ Preoperative immunohistochemistry was not possible in our case because of the small tissue retrieved from the endoscopy-guided biopsy. A boring biopsy, a tissue sampling method that obtains tissue after creating a hole into the submucosa, is an alternative method to obtain more representative tissue compared to a simple biopsy.^14^ The Table describes typical radiologic, histopathologic, and immunohistologic features of GP, along with its differentials, neuroendocrine tumor and gastrointestinal stromal tumor.^2,6,15-17^

**Table.** Radiologic, Histopathologic, and Immunohistologic Features of Gangliocytic Paraganglioma, Neuroendocrine Tumor, and Gastrointestinal Stromal Tumor^2,6,15-17^

	Tumor Type		
Diagnostic Modality	Gangliocytic Paraganglioma	Neuroendocrine Tumor	Gastrointestinal Stromal Tumor
Contrast-enhanced computed tomography	Area of soft tissue attenuation with homogenously enhancing mural or extrinsic tumor	Single/multiple, well-defined intramural or polypoid masses with or without ulceration with contrast enhancement and mesenteric fibrosis and lymphadenopathy	Smooth rounded submucosal mass with mild heterogenous contrast enhancement with necrotic or cystic areas and surrounding organ involvement
Magnetic resonance imaging	Low signal intensity on T1-weighted sequences and high on T2-weighted sequences with homogeneous enhancement on contrast phase	Low signal intensity on T1-weighted sequences and high on T2-weighted sequences; marked homogeneous enhancement on contrast injection	Solid mass with variable signal intensity and intense, heterogenous contrast enhancement
Positron emission tomography (PET)	Solid, well-defined mass with 18F-FDG uptake	Increased uptake of 18F-FDG by high-grade tumors; 18F-FDG PET is not sensitive for low-grade tumors	Used for screening for distant metastases; usually shows FDG-avid lesions but occasionally has poor uptake of 18F-FDG
Histopathology	Three distinct cell types (epithelioid, spindle, and ganglion cells) with varying proportions Epithelioid and spindle cells arranged in a nest and trabecular pattern with ganglion cells scattered in between	Grade 1 and 2 composed of tumor cells possessing round or oval nuclei with salt and pepper chromatin and eosinophilic granular cytoplasm Grade 3 consists of round, ovoid, or spindle-shaped tumor cells with scant cytoplasm or large-cell carcinomas composed of medium or large-sized cells possessing atypical nuclei with evident nucleoli	Spindle cell tumors, often with distinctive extracellular collagen globules Internal cystic component that communicates with the intestinal lumen typical in large tumors (>5 cm)
Immunohistochemistry	Neuron-specific enolase (all 3 cell lines); S100 (mostly in spindle cell component); chromogranin A (mostly in epithelial component); CD56; synaptophysin	Synaptophysin and chromogranin A	DOG-1, CD117, CD34, S100 (focal positivity)

Because of its rarity, no consensus has been established for the management of GP. Patients with local disease and no malignant features or lymph node metastasis on preoperative workup can be considered for endoscopic mucosal resection.^12^ In their systematic review of 263 patients, Okubo et al reported that 10.2% of patients with GP underwent endoscopic mucosal resection, with 1 patient requiring additional surgery.^2^ Surgery is indicated in cases of large tumor size (>2 cm), submucosal extent, periampullary location, pancreatic origin, lymph node metastasis, or suspicion of harboring malignancy.^18^ Local excision is the less radical option that can be considered when lymphadenectomy is not required.^19^ Pancreatoduodenectomy (open/laparoscopic/robotic) is the preferred treatment option when underlying malignancy or lymph node metastasis is suspected. In our case, we performed a robotic-assisted pancreatoduodenectomy because of the lymph node metastasis and a lack of clear preoperative diagnosis. According to our search of the MEDLINE database through November 2022, our case is the first report of GP managed with robotic-assisted pancreatoduodenectomy. The advantages of the robotic approach compared to the laparoscopic approach are 3-dimensional vision, better dexterity of instruments and tremor filtration, and helping to ensure safe performance of surgery with radical lymphadenectomy.

Irradiation and adjuvant chemotherapy do not have a role in the treatment of patients without residual tumors after surgical or endoscopic intervention.^1^ Standard guidelines regarding adjuvant treatment in GP have not been formulated because of their rarity. However, Wong et al reported using intensity-modulated radiotherapy in a case of locally advanced GP.^20^ Recurrence of GP is rare; however, surveillance with annual contrast-enhanced CT of the abdomen is recommended, especially for patients with lymph node metastasis who do not receive adjuvant treatment.^21^

## CONCLUSION

GP, a rare tumor that usually occurs in the duodenum, is often a histologic surprise as most cases are diagnosed in postoperative histopathology. Radical surgical resection is warranted in cases of diagnostic dilemma, suspicion of malignancy, or lymph node metastasis, and robotic-assisted pancreatoduodenectomy is a feasible option. Although the recurrence rate of GP is low, patients need to be followed because of the absence of adjuvant treatment.

## Notes

*This article meets the Accreditation Council for Graduate Medical Education and the American Board of Medical Specialties Maintenance of Certification competencies for Patient Care and Medical Knowledge.*
